# Dectin-1-Mediated Pathway Contributes to *Fusarium proliferatum*-Induced CXCL-8 Release from Human Respiratory Epithelial Cells

**DOI:** 10.3390/ijms18030624

**Published:** 2017-03-13

**Authors:** Chang-Ching Yeh, Huann-Cheng Horng, Hong Chou, Hsiao-Yun Tai, Horng-Der Shen, Shie-Liang Hsieh, Peng-Hui Wang

**Affiliations:** 1 Department of Obstetrics and Gynecology, Taipei Veterans General Hospital, Taipei 112, Taiwan; ccyeh39@gmail.com (C.-C.Y.); hchorng@vghtpe.gov.tw (H.-C.H.); 2 Institute of Clinical Medicine, National Yang-Ming University, Taipei 112, Taiwan; slhsieh@gate.sinica.edu.tw; 3 Department of Obstetrics and Gynecology, National Yang-Ming University, Taipei 112, Taiwan; 4 Institute of BioMedical Informatics, National Yang-Ming University, Taipei 112, Taiwan; 5 Department of Medical Research, Taipei Veterans General Hospital, Taipei 112, Taiwan; chou125@yahoo.com.tw (H.C.); hytai@vghtpe.gov.tw (H.-Y.T.); hdshen@vghtpe.gov.tw (H.-D.S.); 6 Genomics Research Center, Academia Sinica, Taipei 11529, Taiwan; 7 Department of Medical Research, China Medical University Hospital, Taichung 40447, Taiwan

**Keywords:** *Fusarium proliferatum*, CXCL-8, respiratory epithelial cells, Dectin-1

## Abstract

*Fusarium* species are causative agents of human respiratory disorders and are distributed widely in our environment. Little is known of their interaction with human respiratory epithelial cells, which may contribute to allergic airway responses. In this study, we report on the release of C–X–C motif chemokine ligand 8 (CXCL-8) from human bronchial epithelial BEAS-2B cells upon stimulation with *Fusarium proliferatum* extracts. *F. proliferatum*-induced cytokine release from BEAS-2B cells was determined by cytokine array and CXCL-8 enzyme-linked immunosorbent assay (ELISA) kits. Blocking antibodies and signaling pathway inhibitors were employed to delineate cell surface receptors and signaling pathways participating in CXCL-8 release. *F. proliferatum* extracts induced the release of CXCL-8 in a time-dependent manner. The dectin-1 receptor ligands, curdlan and laminarin, reduced CXCL-8 release. Cells pre-treated with anti-Dectin-1 antibodies (2 µg/mL) decreased CXCL-8 release by 24%. Furthermore, *F. proliferatum*-stimulated CXCL-8 release was reduced by 32%, 53%–81%, 40% and 26% after BEAS-2B cells were pretreated with activation inhibitors of spleen tyrosine kinase (Syk)—piceatannol—, mitogen-activated protein kinases (MAPKs)—PD98059, U0126, SB202190, SP600125—, phosphatidylinositol-3-kinase (PI3K)—LY294002—and nuclear factor κ-light-chain-enhancer of activated B cells (NF-κB)—BAY117082—, respectively. These results suggest that Dectin-1-mediated activation of the Syk, MAPKs, PI3K and NF-κB signaling pathways contributes to *F. proliferatum*-stimulated CXCL-8 release from BEAS-2B cells and provides an important basis for developing novel therapeutic strategies in clinical allergy.

## 1. Introduction

Fungi are ubiquitous in our living environment and are important causative agents of human respiratory atopic diseases [1,2,3]. Airway epithelial cells are primordial cells that respond to inhaled potential allergens and irritants and act as a physical barrier, which may also secret mediators that contribute to the pathogenesis of respiratory atopic disorders [4,5,6,7]. However, information regarding interactions between airborne fungi and human airways is still limited.

Airborne fungi such as *Fusarium* may play significant roles in clinical allergy studies [8]. For example, results from Stroud et al. [9] showed that reactivity to fungi was found in approximately 65% of chronic rhinitis patients and reactions to the *Fusarium* (58%), *Alternaria* (39%) and *Pullularia* (38%) species were particularly common. We have previously identified transaldolase as an important allergen of *Fusarium proliferatum* [8], and a vacuolar serine proteinase of *F. proliferatum* has also recently been characterized as a major allergen [10]. However, interactions among *F. proliferatum* and human respiratory epithelial cells are still largely unknown.

We analyzed the cytokine release profile of human bronchial epithelial BEAS-2B cells upon *F. proliferatum* extract stimulation and examined the potential mechanism of C–X–C motif chemokine ligand 8 (CXCL-8) release from these cells. Our results showed that the Dectin-1-mediated pathway, contributes to CXCL-8 release from *F. proliferatum*-stimulated BEAS-2B cells.

## 2. Results

### 2.1. Fusarium proliferatum-Induced Cytokines Release by BEAS-2B Cells

**Figure 1:**
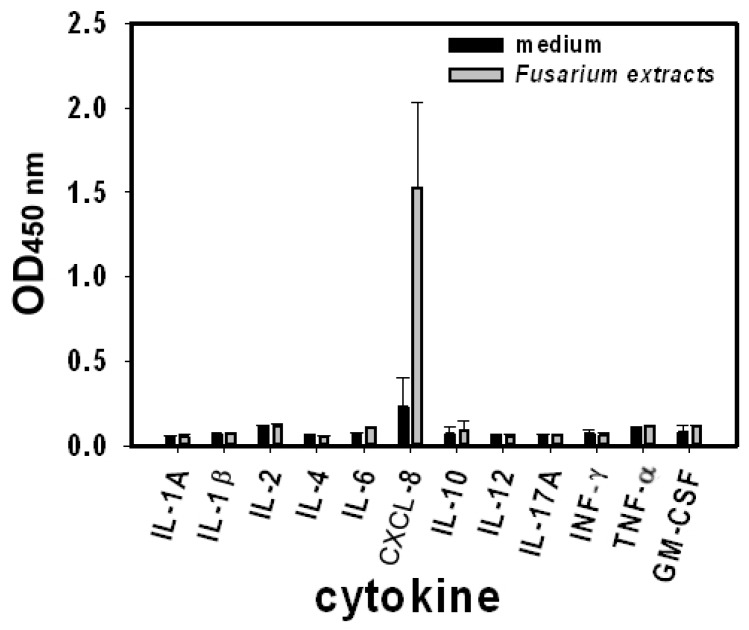
*Fusarium proliferatum-*induced cytokines released from human lung epithelial BEAS-2B cells. BEAS-2B cells were stimulated with *F. proliferatum* extracts (100 µg/mL) and were incubated for 24 h before cytokine release in the supernatants was analyzed with a Cytokines Multi-Analyte ELISArray Kit. Data shown here represent two independent experiments performed in duplicate and are presented as the mean optical density (OD) at 450 nm of two experiments ± standard deviations. IL: Interleukin; CXCL-8: C–X–C motif chemokine ligand 8; INF-γ: Interferon γ; TNF-α: Tumor necrosis factor α; GM-CSF: granulocyte-macrophage colony-stimulating factor.

To investigate the profile of cytokine release from human bronchial epithelial cells in response to *F. proliferatum* stimulation, BEAS-2B cells were exposed to *F. proliferatum* extracts (100 µg/mL) for 24 h. The concentration of interleukin (IL)-1A, IL1-β, IL-2, IL-4, IL-6, IL-8/CXCL-8, IL-10, IL-12, IL-17A, interferon γ (INF-γ), tumor necrosis factor α (TNF-α) and granulocyte-macrophage colony-stimulating factor (GM-CSF) in cultured supernatants was analyzed with a Cytokines Multi-Analyte ELISArray Kit. Compared to the control cells, BEAS-2B cells treated with *F. proliferatum* extracts showed a significant increase in CXCL-8 release (Figure 1). No significant differences were observed for the release of other mediators between treated and unexposed cells (Figure 1). CXCL-8 is an important chemotactic cytokine in human lung inflammation, therefore, we focused on the mechanism of CXCL-8 release from BEAS-2B cells.

**Figure 2:**
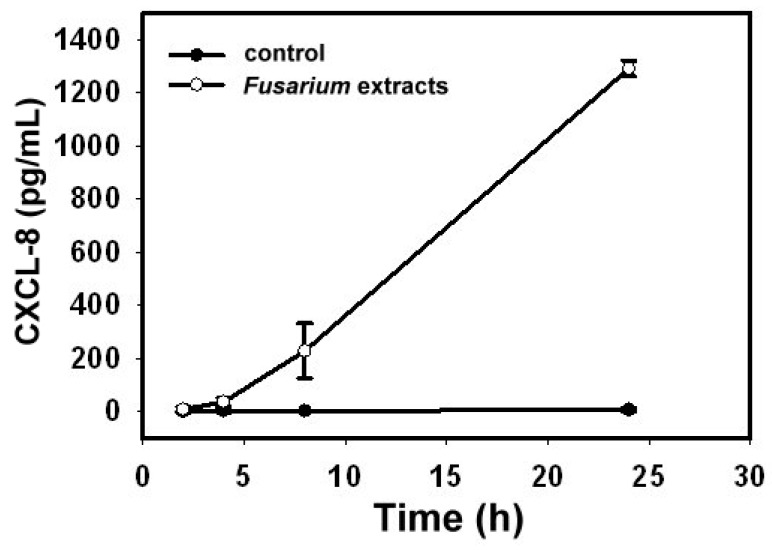
*F. proliferatum* induces CXCL-8 release in a time-dependent manner. BEAS-2B cells were treated with *F. proliferatum* extracts (100 µg/mL) for 2, 4, 8 and 24 h. The level of CXCL-8 in the culture medium was determined by enzyme-linked immunosorbent assay (ELISA). The data shown represent three independent experiments performed in duplicate and are presented as the mean of three experiments ± the standard deviations.

BEAS-2B cells were exposed to *F. proliferatum* extracts (100 µg/mL) and CXCL-8 release was examined at 2, 4, 8 and 24 h intervals. Compared to un-stimulated cells, the level of CXCL-8 in the cultured supernatant was elevated as early as 4 h after exposure to *F. proliferatum* extracts and continued to increase (Figure 2).

BEAS-2B cells were also exposed to *F. proliferatum* extracts at concentrations ranging from 50 to 150 µg/mL for 24 h. Our results showed that *F. proliferatum* induced CXCL-8 release in a dose-dependent manner [11].

### 2.2. The Role of β-Glucan Structure in Fusarium proliferatum-Induced CXCL-8 Release by BEAS-2B Cells

**Figure 3:**
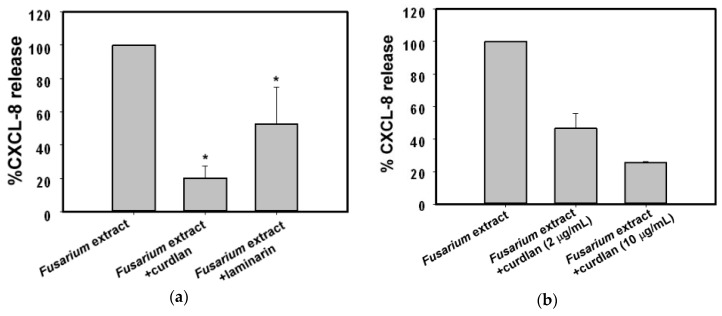
Effect of β-glucan on CXCL-8 release. (**a**) Curdlan (10 µg/mL) or laminarin (10 µg/mL) was added to BEAS-2B cells for 1 h prior to stimulation with 100 µg/mL of *F. proliferatum* extracts for 24 h. The supernatant was collected and the secretion of CXCL-8 determined by ELISA; (**b**) BEAS-2B cells were pre-incubated with curdlan at the indicated concentrations for 1 h. Cells were then exposed to *F. proliferatum* extracts for 24 h and the amounts of CXCL-8 in the cultured supernatants were determined by ELISA. The results are representative of three (**a**) and two (**b**) independent experiments performed in duplicate. Asterisks (*) indicate significant differences (*p* < 0.05) between the paired samples.

β-Glucan is the main component of fungal cell walls. In this study, we focused on the role of *Fusarium*-derived β-glucan moieties interacting with BEAS-2B cells. Laminarin (10 µg/mL) and curdlan (10 µg/mL), β-glucan receptor ligands, were pre-incubated individually with BEAS-2B cells for 1 h before stimulating with *F. proliferatum* extracts. We found that curdlan and laminarin significantly reduced CXCL-8 release from *F. proliferatum*-treated BEAS-2B cells to 20% and 53% of the controls without incubation with inhibitors, respectively (Figure 3a). Incubation of BEAS-2B cells with curdlan or laminarin alone did not affect CXCL-8 release (data not shown). Furthermore, two different concentrations of curdlan (2 or 10 µg/mL) were used in the inhibition studies. The data (Figure 3b) showed that curdlan decreased *F. proliferatum* extracts-induced CXCL-8 release to 46% and 25% of the controls without incubation with inhibitors, respectively (Figure 3b).

### 2.3. Role of Dectin-1/Spleen Tyrosine Kinase in the Fusarium proliferatum Extract-Induced CXCL-8 Release

**Figure 4:**
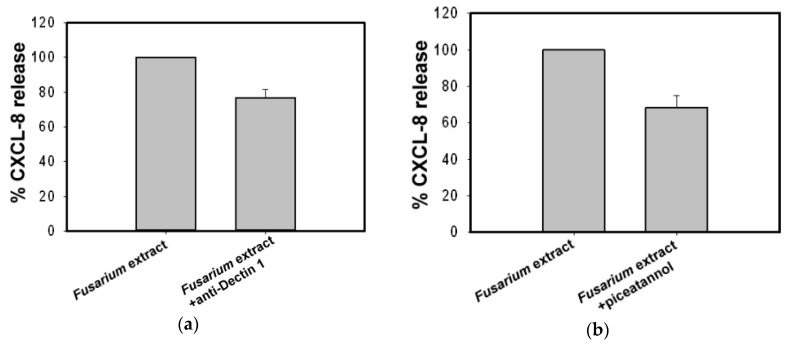
Effect of the anti-Dectin 1 and Syk specific inhibitor piceatannol on *F. proliferatum* stimulated CXCL-8 release. BEAS-2B cells were pre-incubated in the absence or presence of mouse monoclonal antibody against human Dectin-1 (2 µg/mL) (**a**) or Syk inhibitor piceatannol (5 μM) (**b**) for 1 h. Cells were then exposed to *F. proliferatum* extracts for 24 h and cultured supernatants were collected for CXCL-8 determination by ELISA. Results are representative of three independent experiments performed in duplicate.

Curdlan has been identified as a Dectin-1 specific ligand [12]. To determine whether a Dectin-1 like receptor is involved in the *F. proliferatum*-induced CXCL-8 release, we used antibodies against human Dectin-1 to examine its effect on CXCL-8 release. BEAS-2B cells were pre-treated with anti-Dectin-1 (2 µg/mL) for 1 h before exposure to *F. proliferatum* extracts for 24 h. Results showed that CXCL-8 release decreased by 24% compared to that of cells without antibody pre-treatment (Figure 4a).

Spleen tyrosine kinase (Syk) is involved in Dectin-1 signaling and plays an important role in antifungal immune responses [13,14,15]. To assess whether CXCL-8 release from BEAS-2B cells exposed to *F. proliferatum* extracts is dependent on Syk signaling, a Syk inhibitor was used to examine its contribution to this pathway. BEAS-2B cells were incubated with *F. proliferatum* extracts in the presence or absence of 5 μM piceatannol. Results shown in Figure 4b indicate that the Syk inhibitor reduced *F. proliferatum* extracts-induced CXCL-8 secretion by 32%.

### 2.4. Involvement of the Mitogen-Activated Protein Kinases, phosphatidylinositol-3-kinase and Nuclear Factor κ-Light-Chain-Enhancer of activated B cells in Fusarium proliferatum-Induced CXCL8 Release

**Figure 5:**
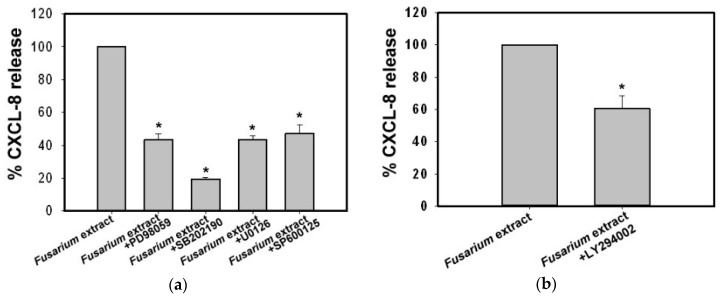
Effects of mitogen-activated protein kinases (MAPKs) and phosphatidylinositol-3-kinase (PI3K) inhibitor on CXCL-8 release by *F. proliferatum*-stimulated BEAS-2B cells. BEAS-2B cells were pre-treated in the presence or absence of MAPKs inhibitors PD98059 (MEK1 inhibitor, 10 μM), SB202190 (p38 inhibitor, 10 μM), U0126 (ERK1/2 inhibitor, 10 μM), and SP600125 (JNK inhibitor, 5 μM) (**a**) and PI3K inhibitor LY294002 (5 μM) (**b**), respectively, for 1 h before exposure to *F. proliferatum* extracts (100 µg/mL) for 24 h. Supernatant was collected and the level of CXCL-8 was determined by ELISA. Results are representative of two independent experiments performed in triplicate. Asterisks (*) indicate significant differences (*p* < 0.05) between the paired samples.

To evaluate whether the mitogen-activated protein kinases (MAPKs) signaling pathways are involved in the *F. proliferatum*-induced CXCL-8 release, we examined the effect of inhibitors on three subfamilies of MAPKs. Cells were pre-incubated individually, with various inhibitors prior to stimulation with *F. proliferatum* extracts. As shown in Figure 5a, SB202190, a p38 MAPK inhibitor, exhibited the strongest effect and reduced the *F. proliferatum*-induced CXCL-8 release by 81% at 10 μM (*p* < 0.01). At the same concentration, the MEK1 inhibitor PD98059 and the ERK1/2 inhibitor U0126 reduced the CXCL-8 release by 57%. The c-Jun NH2-terminal kinase (JNK) inhibitor SP600125 inhibited 53% of CXCL-8 release at 5 μM (Figure 5a). Without the *F. proliferatum* extracts, none of the inhibitors had any effect on CXCL-8 release (data not shown). These results indicate that ERK1/2, p38 and JNK pathways are involved in CXCL-8 release from BEAS-2B cells stimulated with *F. proliferatum* extracts.

In addition to MAPKs, a number of protein kinases were also involved in the release of mediators. phosphatidylinositol-3-kinases (PI3K) and its downstream components have been shown to play a prominent role in various inflammatory cells by controlling cell growth, differentiation, survival, proliferation, and cytokine production [16]. In this study, we examined the contribution of PI3K to CXCL-8 release from BEAS-2B cells. The data showed that LY294002 (5 μM), a PI3K inhibitor, reduced CXCL-8 release by 40% (Figure 5b). This result implies that *F. proliferatum* extracts activate the PI3K pathway.

**Figure 6:**
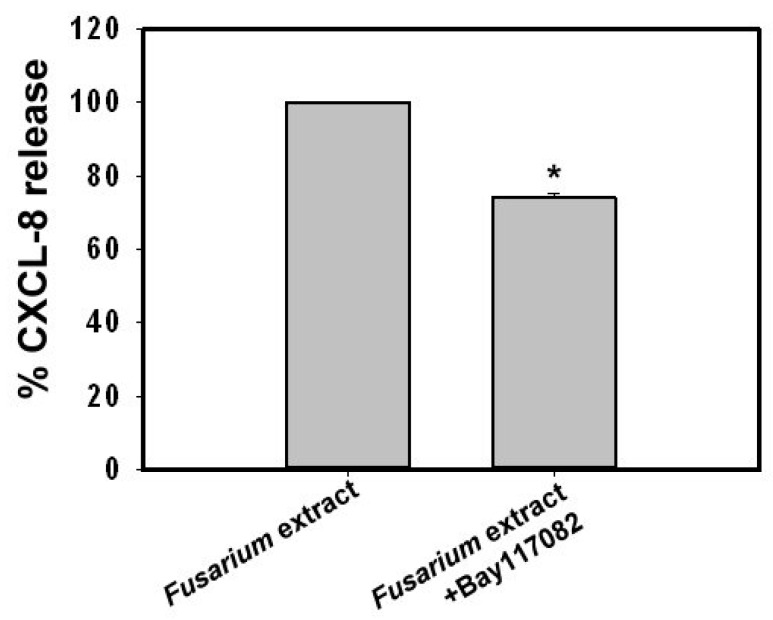
Effect of nuclear factor κ-light-chain-enhancer of activated B cells (NF-κB) inhibitor on *F. proliferatum*-stimulated CXCL-8 release. BEAS-2B cells were pre-incubated with BAY117082 (5 μM) for 1 h before treating with *F. proliferatum* extracts (100 µg/mL) for 24 h. Cultured supernatants were collected and the level of CXCL-8 was determined by ELISA. The results are representative of three independent experiments performed in triplicate. Asterisks (*) indicate significant differences (*p* < 0.05) between the paired samples.

Studies on Dectin-1, Dectin-2 and Mincle have revealed that several signaling components of C-type lectin receptors can induce nuclear factor κ-light-chain-enhancer of activated B cells (NF-κB) activation [17]. To investigate whether *F. proliferatum* extracts-induced CXCL-8 release is mediated via NF-κB activation, BEAS-2B cells were stimulated with *F. proliferatum* extracts in the presence or absence of IκB phosphorylation inhibitor BAY117082 (5 μM). BAY117082 reduced 26% of CXCL-8 release at 5 μM. Thus, NF-κB is, at least partially involved in CXCL-8 release from *F. proliferatum*-treated BEAS-2B cells (Figure 6).

## 3. Discussion

Although *Fusarium* is one of the prevalent airborne fungi in our environment [18], not much is known about the role of *Fusarium* species in clinical allergy. Results from the present study showed that stimulation of a human lung epithelial cell line, BEAS-2B cells, with *F. proliferatum* extracts induces a strong release of the pro-inflammatory and chemotactic factor CXCL-8 in a time-dependent manner (Figure 1 and Figure 2).

During the course of a human cell and fungal interaction, multiple host pattern recognition receptors are likely to be stimulated by different fungal pathogen-associated molecular patterns in different combinations depending on the host cell types and on the fungal species [19]. β-glucans are a major component of fungal cell walls [20,21]. Studies have indicated that β-glucan may be a target for immune recognition by airway epithelial cells and induces cytokine release via β-glucan dependent pathways [22,23,24]. Recently, the β-glucan receptor Dectin-1 has been shown to be expressed in human primary normal bronchial epithelium cells and an A549 cell line [25,26]. In this study, our results showed that *F. proliferatum* extracts-induced CXCL-8 release can be inhibited by Dectin-1 receptor ligands curdlan and laminarin (Figure 3). Curdlan is a linear (1→3) β-glucans and laminarin is composed of (1→3) (1→6)-β-glucan linkages. Laminarin is a water soluble small (6–8 kDa) linear β-glucan with a degree of polymerization of 20–30 which can bind to Dectin-1 without stimulation of downstream signaling [27,28,29,30]. Nevertheless, it can block the binding of larger β-glucans such as zymosan to Dectin-1 [28], as well as suppress the expression of IL-6 and TNF-α induced by curdlan at both mRNA and protein levels [30]. In this study, laminarin at 10 µg/mL was unable to stimulate CXCL-8 release from BEAS-2B cells alone (data not shown); however, it reduced CXCL-8 release from *F. proliferatum*-treated BEAS-2B cells to 53% of the controls without incubation with inhibitors (Figure 3). In contrast, curdlan is a long β-d-glucan with a degree of polymerization of ~500 [29]. It was found to stimulate mRNA expression of TNF-α and IL-6 in a dose-dependent manner, such as in cultured human corneal epithelial cells [30]. In our preliminary study, curdlan at 100 µg/mL was shown to stimulate CXCL-8 release from BEAS-2B cells (data not shown). Therefore, curdlan at 10 µg/mL which was unable to stimulate CXCL-8 release from BEAS-2B cells (data not shown) was used in this study. We found that curdlan (10 µg/mL) significantly reduced CXCL-8 release from *F. proliferatum*-treated BEAS-2B cells to 20% of the controls without incubation with inhibitors (Figure 3). Although we did not determine β-glucans in *F. proliferatum* extracts, the results obtained suggest that there may be β-glucan structures or some other as yet unidentified *Fusarium*-derived agents in *F. proliferatum* extracts, which may contribute to receptor binding and interaction with human airway epithelial cells. With respect to curdlan and laminarin, the Dectin-1 antibody showed less inhibitory effect (Figure 4a). One possible explanation is that *F. proliferatum* extracts bind Dectin-1 like receptors and others on BEAS-2B cells. Currently, we cannot exclude the possibility that the anti-Dectin-1 used was of low affinity.

Ligation of Dectin-1 by fungal β-glucan leads to Syk activation [13,14,15,31]. Our results show that Syk inhibitor piceatannol (5 μM) can diminish *F. proliferatum*-induced CXCL-8 secretion by 32%. This suggests that interactions between β-glucan moieties and Dectin-1 receptors in BEAS-2B cells result in CXCL-8 release.

To further explore molecular mechanisms contributing to *F. proliferatum*-induced CXCL-8 release, specific inhibitors that block MEK/ERK, p38MAPK, JNK, PI3K and NF-κB activation pathways were used. Our results indicated that PD98059 and U0126 (MEK/ERK inhibitors), SB202190 (p38 MAPK inhibitor) and SP600125 (JNK inhibitor) significantly reduced (>50%) CXCL-8 release induced by *F. proliferatum* extracts. In contrast, BAY117082 (IκB inhibitor) was less potent (26% reduction). This suggests that MEK/ERK, p38 MAPK, JNK and NF-κB pathways are all involved in CXCL-8 release when BEAS-2B cells are stimulated with *F. proliferatum* extracts. In addition, PI3K was also observed to be involved in CXCL-8 release, and indicates that *F. proliferatum* extracts induce CXCL-8 release via multiple signaling pathways. Based on these results, it can be said that innate receptors and signaling pathways contribute to *F. proliferatum-*induced CXCL-8 release from BEAS-2B cells.

Results obtained from this study indicate that Dectin-1-dependent pathways contribute to *F. proliferatum*—stimulated pro-inflammatory CXCL-8 chemotactic factor release from lung epithelial BEAS-2B cells. House dust mites (HDM) have been identified as the most important allergens responsible for human atopic diseases worldwide. Interestingly, results from Nathan et al. [24] also suggested that β-glucan receptors, such as Dectin-1, mediates the activation of the airway epithelium via CCL20 secretion by HDM and may link innate pattern recognition at the airway surface with adaptive immune responses. Fungal spores are ubiquitous in our environment and β-glucans are a major component of fungal cell walls. Results obtained from this study and others suggest that Dectin-1-mediated signaling (which may be induced through environmental β-glucan structures or some other as yet unidentified agents exposure including those from fungi and house dust mite) may contribute to the initiation of early allergic airway responses and trigger atopic exacerbation. These findings provide the basis for developing novel therapeutic strategies in clinical allergy.

## 4. Materials and Methods

### 4.1. Materials

Curdlan, laminarin, piceatannol, Bay117082 and SP600125 were purchased from Sigma-Aldrich (Chemical Co., St. Louis, MO, USA); PD98059 and U0126 were purchased from Cell Signaling Technology (Beverly, MA, USA); SB202190 was purchased from Calbiochem (Merck KGaA, Darmstadt, Germany); and mouse anti-human Dectin 1 was purchased from R & D systems, Inc. (Minneapolis, MN, USA).

### 4.2. Crude Extracts of Fusarium proliferatum

The *F. proliferatum* strain BCRC 30972 used in this study was isolated from the air of Taiwan and provided by the Food Industry Research and Development Institute, Hsinchu, Taiwan. It was cultured in a CYB medium, without agitation at 26 °C for 5 days. The CYB medium contains a yeast carbon base (Difco Laboratories, Detroit, MI, USA; 11.7 g/L), glucose (Mallinckrodt Baker, Inc., Phillipsburg, NJ, USA; 10 µg/L) and casein enzymatic hydrolysate (Sigma-Aldrich; 10 µg/L). Fungal spores and mycelia were collected and ground under liquid nitrogen with a mortar and pestle and the crude extracts were prepared essentially as described in References [8,10,32]. The protein content of crude fungal extracts was determined with a dye-binding assay according to the manufacturer’s instructions (Bio-Rad, Richmond, CA, USA).

### 4.3. Cell Culture and Treatment

Human bronchial epithelial BEAS-2B cell line (bronchial epithelia, adenovirus 12-SV40 transformed) was obtained from the American Type Culture Collection (ATCC, Manassas, VA, USA). BEAS-2B cells were cultured at 37 °C in an atmosphere of 5% CO_2_ in RPMI-1640 (Life Technologies Gibco BRL, Grand Island, NY, USA), supplemented with 10% heat-inactivated fetal calf serum (Hyclone, Logan, UT, USA) and antibiotics (penicillin 100 U/mL, streptomycin 100 µg/mL; Gibco).

BEAS-2B cells were grown in 24-well culture plates (Corning Costar, Corning, NY, USA) to 80%–90% confluency. Cells were washed three times with Hank’s balanced salt solution (Sigma–Aldrich) before treating with *F. proliferatum* extracts. For mechanism studies, cells were pre-treated with specific inhibitors for 1 h, including PD98059 (10 μM), SB202190 (10 μM), U0126 (10 μM), SP600125 (5 μM), LY294002 (5 μM), BAY117082 (5 μM), piceatannol (5 μM), and then treated with *F. proliferatum* extracts (100 µg/mL) for 24 h in a serum-free medium. In polysaccharide and antibody-blocking experiments, β-glucan moieties (curdlan 10 µg/mL, laminarin 10 µg/mL) or mouse anti-human Dectin-1 antibodies (2 µg/mL) were used as competitive inhibitors. Cell culture supernatants were collected and stored at −80 °C for cytokine assays.

### 4.4. Cytokine Array and CXCL-8 ELISA Assay

*F. proliferatum*-stimulated cell culture supernatants were analyzed for IL-1A, IL1-β, IL-2, IL-4, IL-6, CXCL-8, IL-10, IL-12, IL-17A, TNF-α, IFN-γ, and GM-CSF using a Human Inflammatory Cytokines Multi-Analyte ELISArray™ Kit (SABiosciences, Frederick, MD, USA) according to the manufacturer’s instructions. The level of CXCL-8 was measured using a commercially available ELISA kit (R & D Systems) in accordance with the manufacturer’s instructions.

### 4.5. Statistical Analyses

Data were presented as mean ± SD from at least two independent experiments. Statistical analysis of the data was performed using Student’s *t* test and values were considered statistically significant when the *p*-value was <0.05.

## 5. Conclusions

Results obtained from this study suggest that the β-glucan moieties or some other as yet unidentified agents of *F. proliferatum* interact with the Dectin-1 receptor of airway epithelial cells and activate the Syk, MAPKs, PI3K and NF-κB signaling pathways to induce CXCL-8 release. This may contribute to the initiation of early allergic airway responses and trigger atopic exacerbation and provides an important basis for developing novel therapeutic strategies in clinical allergy.

## Abbreviations

Syk	Spleen tyrosine kinase
MAPKs	Mitogen-activated protein kinases
PI3K	Phosphatidylinositol-3-kinase
HDM	House dust mite
